# Competition, Cooperation, and the Mechanisms of Mental Activity

**DOI:** 10.3389/fpsyg.2018.01352

**Published:** 2018-08-03

**Authors:** Carlos Blanco-Pérez

**Affiliations:** Department of Philosophy, Universidad Pontificia Comillas, Madrid, Spain

**Keywords:** competition, cooperation, neural architecture, mental functioning, selection

## Instruction, selection, and cooperation

Understanding the nature of mental function represents one of the deepest challenges for contemporary neuroscience. Although the precise manner in which different cerebral structures enable higher-order cognitive processes (the meticulous path leading “from molecules to mind”) is still a mystery, a number of influential models of mental function have stressed the importance of competition between neural circuits as one of the main mechanisms behind behavioral flexibility and the sophistication exhibited by higher-order cognitive processes. Concepts like “selective stabilization of synapses” (Changeux et al., 1973; Changeux and Danchin, 1976) and “neural Darwinism” (Edelman, 1993), which can be encompassed into a family of explanatory models based upon variation/selection strategies, try to understand how the interaction with the environment affects neural architecture, by reshaping the fine structure of synaptic connections.

The relevance of selective mechanisms for understanding mental processes cannot be underestimated. However, in these models it is sometimes difficult to identify the exact units of replication (particularly if one wants to adhere to a strictly Darwinian paradigm) and the selective criteria employed by the brain, at least in absence of reproductive arguments of the kind present in standard biological Darwinism. Also, notwithstanding the widespread lack of pre-specification and the high degrees of versatility that may be attributed to neuronal groups in Edelman's model, it is clear that cortical functions are on many occasions located in certain areas (Blanco Pérez, 2017). Hence, the problem of how to reconcile localization and integration still remains unsolved to a large extent.

Furthermore, at a theoretical level it is also necessary to take into consideration the effects of cooperation between neural circuits and the way in which this process is related to competition. Of course, the crucial role of cooperation is also acknowledged by the most influential selective models of cognitive function, in which the combination of genetics and selection is often channeled through cooperative strategies bridging among the different levels of brain organization, in accordance with the adaptive consequences of a certain behavior. Nevertheless, it is worth addressing some important features of cooperative mechanisms that may suggest their conceptual independence.

The specificity of neuronal connections cannot be explained neither by a one-to-one biochemical matching stemming from a genetic program nor by selective stabilization derived from the acquisition of new experiences in an often unpredictable environment. While these processes may suffice to justify the existence and prevalence of certain processing routes, they fail to explain how information is integrated into a unified percept. If, in highly simplified terms, genetic and selective routes account for the degree of specialization manifest in some neural processes, in which sensory information is carefully discriminated, at some levels of brain organization they must also favor cooperative strategies that introduce additional degrees of variability or “non-specialization.” This flexibility would lie at the basis of the ability to unify heterogeneous features into a coherent perceptual whole, a capacity that seems to constitute one of the fundamental characteristics of mental function.

Cooperation can be understood in terms of the different mechanisms of cellular communication between groups of neurons and larger networks. One of them is the so-called “Hebbian learning,” according to which synaptic connections are strengthened when the neurons involved have highly correlated outputs. At a neural scale, this process entails the possibility that circuits processing information of a convergent, yet heterogeneous nature may establish neuronal networks. Communication between circuits helps us understand the unification of the various properties of objects into a single percept. The possibility of communication is perhaps best defined by physical aspects like the proximity between circuits that are topically organized, the nature of the sensorial system itself, and the ability of certain neuronal networks to build common patterns of activity that facilitate the convergence between different kinds of information. Thus, hypothetically we can expect that both proximity across space and temporal synchronization will elicit certain patterns of wiring in neuronal networks. The nature and properties of the stimulus are instantiated in the pathways themselves, that is to say, in the structural organization of the particular sensory system.

Such a process of integration must take place at different scales. Broadly speaking, the first level would be given by the capacity of a certain sensory pathway to distill information from the stimulus. At a second, multilevel stage, unification would happen within the sensory system as a whole (like the visual system), in order to obtain a unified perception within that sensory modality. But it is still possible to theorize about a higher level of integration: the perceptual whole, in which different sensory modalities converge into a coherent unity (the mystery of integration, elegantly summarized by Karl Lashley: “how [is it that] the specialized areas of the cerebral cortex interact to produce the integration evident in thought and behavior”) (Lashley, 1931). This ability is related to large-range connectivity, especially if one takes into account that “no area of the cerebral cortex connects with only one or two other areas” (Zeki and Shipp, 1988).

If selective strategies analyze and decompose features of the stimulus through functional differentiation and hierarchical division of labor, horizontal processes help synthesize this information into a coherent whole. In more abstract terms, the mechanisms of functional differentiation represent successful evolutionary strategies for dealing with the complexities of the environment. However, those processes that tend to integrate those differences across space and time into unified percepts are the necessary counterpart to the dangers posed by growing degrees of specialization, which could obstruct the perception of reality as a whole.

Hence, from this perspective the functional logic of cortical connections implies the coexistence of genetically determined, competitive, and cooperative processes, built upon operating principles of different conceptual nature. The final wiring patterns that define neuronal connections should then be described by at least three classes of processes: genetic mechanisms that instruct the creation of certain neuronal assemblies (through gene networks that code for neuronal networks; Greenspan, 2009), selective epigenetic strategies that follow neuronal activity and reshape previously formed networks, and cooperative epigenesis based upon the possibility of communication between different networks. This combination of instruction, selection, and cooperation grants the brain an exuberance of configurative possibilities and may be regarded as the essential underpinning of its behavioral versatility.

## Some sensory systems

As it is well known, visual information is channeled through different routes, each of which has achieved an outstanding degree of functional specialization in treating certain features of the visual stimulus, like motion, form, and color (Zeki and Shipp, 1988). The principle of functional specialization in the visual cortex is therefore of the highest importance for understanding the nature of perception. Information concerning color is processed in the V4 area, whereas information about motion pertains to the V5 system, such that damage in the first region (like achromatopsia) may not affect the second system (one of whose most notorious pathologies is akinetopsia; Zeki, 2003).

Specialization, regarded as the division of neural work between groups of cortical neurons, is governed by a hierarchical processing of information, in which it is possible to attain growing levels of abstraction (or “formalization”) in the assimilation of the stimulus. In ascending pathways, functional differentiation thus coexists with cooperation between the different groups of cells.

Given that information in sensory systems is processed in parallel and in hierarchical ways, through relay cells, functional differentiation is guaranteed across thalamo-cortical and cortico-cortical communication. From sensory periphery to higher brain centers (Guillery and Sherman, 2002) complex cells—like pyramidal neurons of great size—control the activity of groups of simpler nerve cells, in a relay chain running across stellate cells in the 4C layer, ganglion cells, and photoreceptors.

Thus, the functionality shown by the visual system is enabled by certain notable anatomic features. Among them it is worth remarking its hierarchical organization (which favors growing degrees of abstraction from the content of the stimulus), the presence of parallel routes (each of which has acquired important levels of specialization and *divide et impera* strategies, while at the same time guaranteeing convergence between these differentiated pathways), the topographic organization of the system, and the existence of vertical columns of nerve cells that gather neurons endowed with similar properties (Hubel and Wiesel, 1968). Likewise, the analysis of such structural characteristics needs to be merged with that of the activity of the neural circuits operating in this sensory system, in particular the feedback neuronal responses via reentrant or recursive connections.

## The unification of mental function

At this point one can appreciate the necessity of combining the study of the basic molecular mechanisms with the approach inspired by systems neuroscience, such that bottom-up and top-down processes may converge into a unified picture of the brain and its related functions. Indeed, it seems clear that understanding the brain as a whole demands the integration of reductionist and holistic frameworks. For example, deciphering the fundamental chemical mechanisms offers the possibility of gradually unveiling the vast thread that leads from genes, neurons, messengers, glia, and synapses to mental function as a whole.

In this dynamic interplay between inherited and acquired elements of information, and, moreover, between specificity and plasticity (Merzenich et al., 1988), through which activity and experience can remodel cerebral function, it is possible to identify one of the most powerful adaptive features of the brain. While the fundamental neuroanatomical structures may enjoy high degrees of stability (defined by a genetic program of instructions, applied from early natal and postnatal development through critical periods), functional connectivity shows a remarkable level of plasticity. Equilibrium between structural stability and functional mutability is attained through a process of constant regulation, in which nerve cells are capable of producing consistent patterns of activity, susceptible to modification in accordance with the new experiences accumulated by the subject. Stemming from instructive mechanisms, bottom-up causal lines can be interpreted as constraints that limit the overall variability of the system; however, the existence of top-down processes enables the amplification of its variability within the boundaries set by those constraints.

Lack of pre-specification in a significant number of neuronal connections offers a powerful tool for adaptability to a changing environment. Rather than creating rigid patterns that bind neurobiological structures and functions in unique forms, the brain has developed an efficient way of coping with environmental challenges through a highly flexible representational architecture (Dehaene-Lambertz et al., 2002).

Along these lines, the difficult interplay between, on the one hand, localization (which is an approach that tends to regard mental activity as essentially codified in the activity of specific neural connections) and, on the other hand, integration (according to which behavior and mental activity stem from the integrated activity of the brain) needs to pay attention to processes of functional specialization, mediated by selective mechanisms, and processes of functional integration. The latter require mechanisms of cooperation between neural networks, many of which may indeed be channeled through specific organs in charge of connecting physically separated regions of the brain or distant zones within a certain region.

This ISC model (instruction-selection-cooperation) can be summarized as the combination of instruction, capable of creating a stable but relatively flexible neuronal architecture, selection through interaction with the environment, and cooperation, which would play a fundamental role in promoting the unification of mental function visible in the integrated nature of many perceptions and behaviors. Thus, the unrolling of Ariadna's subtle thread that connects the most elementary neurobiological structures with the higher cognitive functions would demand the proper integration of these three basic classes of processes. From a metaphorical point of view, “selfish circuits” must coexist with “altruistic networks.”

## Author contributions

The author confirms being the sole contributor of this work and approved it for publication.

### Conflict of interest statement

The author declares that the research was conducted in the absence of any commercial or financial relationships that could be construed as a potential conflict of interest.
